# Radiofrequency ablation versus hepatic resection for recurrent hepatocellular carcinoma: an updated meta-analysis

**DOI:** 10.1186/s12876-020-01544-0

**Published:** 2020-11-27

**Authors:** Daopeng Yang, Bowen Zhuang, Yan Wang, Xiaoyan Xie, Xiaohua Xie

**Affiliations:** Department of Medical Ultrasonics, Institute of Diagnostic and Interventional Ultrasound, The First Affiliated Hospital of Sun Yat-Sen University, NO.58 Zhongshan Road 2, Guangzhou, 510080 People’s Republic of China

**Keywords:** Hepatocellular carcinoma, Recurrent, Radiofrequency ablation, Hepatic resection, Meta-analysis

## Abstract

**Background:**

The clinical benefits of treatment with radiofrequency ablation (RFA) and repeat hepatic resection (RHR) for recurrent hepatocellular carcinoma (RHCC) remain controversial. This meta-analysis aims to evaluate the outcomes and major complications of RFA versus RHR in patients with early-stage RHCC.

**Methods:**

PubMed, Embase, Web of Science and the Cochrane Library were systematically searched for comparative studies on the evaluation of RHR versus RFA for RHCC. The primary outcome was overall survival (OS), and the secondary outcomes were progression-free survival (PFS) and major complications. Meta-analysis was performed using a random-effects model or fixed-effects model, and heterogeneity was tested by the Cochran Q statistic.

**Results:**

Ten studies with 1612 patients (RHR = 654, RFA = 958) were included in the meta-analysis. The meta-analysis showed that RHR had superior OS (HR 0.77, 95% CI =0.65–0.92, *P* = 0.004) and PFS (HR 0.81, 95% CI =0.67–0.98, *P* = 0.027) compared to RFA, whereas major complications may be less frequent in the RFA group (OR 0.15, 95% CI = 0.06–0.39, *P* < 0.001). In the subgroup analysis of patients with single RHCC ≤3 cm, OS (HR 1.03, 95% CI =0.69–1.52, *P* = 0.897) and PFS (HR 0.99, 95% CI = 0.71–1.37, *P* = 0.929) showed no significant differences in the comparison of RHR and RFA. In single RHCC> 3 cm and ≤ 5 cm, RFA showed an increased mortality in terms of OS (HR 0.57, 95% CI = 0.37–0.89, *P* = 0.014).

**Conclusion:**

RHR offers a longer OS and PFS than RFA for patients with RHCC, but no statistically significant difference was observed for single RHCC ≤3 cm. The advantages of fewer major complications may render RFA an alternative treatment option for selected patients.

## Background

Hepatocellular carcinoma (HCC) is the fifth most common cancer and the third most common cause of cancer-related death worldwide [1]. Although hepatic resection(HR) remains a curative treatment for HCC [2], the long-term outcomes after resection are not yet satisfactory, as the incidence of tumor recurrence can be up to 60–80% within 5 years [3, 4], and the reported 5-year survival rate of HCC ranges from 40 to 50% [5–7]. Currently, repeat hepatic resection (RHR) and radiofrequency ablation (RFA) are available as the major curative treatments for early-stage recurrent hepatocellular carcinoma (RHCC) [8–10]. RHR is typically considered the first-line treatment for RHCC [7], but its application is limited by more limited liver function reserve and technical difficulties than initial resection [11, 12]. Compared with RHR, RFA has advantages in high repeat applications and fewer complications. Thus, RFA is considered safer with less damage in treating RHCC following primary resection [13, 14]. Though some studies have compared the clinical outcomes of RHR versus RFA in RHCC, the results remain controversial. Several studies have shown that the outcomes following RFA are similar to those following RHR in the treatment of RHCC [15, 16]. In contrast, some reports have demonstrated that RHR provides a survival advantage in RHCC compared with RFA [17, 18]. However, these comparative studies were limited by the small number of cases and potential confounding factors. To date, 4 meta-analyses have been published to explore the outcomes of RFA versus RHR in RHCC [8–10, 19]. However, the evidence for these studies was poor because of the few included studies and the lack of randomized controlled trials (RCTs). Recently, several articles, including an RCT, have been published, which have never been included in previous meta-analyses [17, 18, 20–22]. Therefore, our meta-analysis aimed to compare the efficacy and safety of RHR and RFA in early-stage RHCC by adding the latest published comparable studies.

## Method

Institutional review board approval was not required for this type of study at the authors’ institutions. The study adhered to the Preferred Reporting Items for Systematic Reviews and Meta-Analyses (PRISMA) statement [23] (Supplementary Table 1). The study was preregistered with the International Prospective Register of Systematic Reviews (PROSPERO; reg. no. CRD42020172689).

### Search strategy

We systematically searched PubMed, Embase, Web of Science and the Cochrane Library for articles published from inception to March 1, 2020, on the application of RFA and HR in the treatment of RHCC. The search terms included the terms related to RFA, hepatic resection, and RHCC (Supplementary Table 2). Only studies on humans and in English were considered for inclusion. The reference lists of all potentially useful relevant articles were searched to identify additional articles for inclusion.

### Inclusion and exclusion criteria

The inclusion criteria were as follows: 1. clinical or pathological diagnosis of RHCC; 2. recurrent HCC patients undergoing surgical resection and patients in the control group undergoing RFA; 3. no evidence of macroscopic vascular invasion or extrahepatic distant metastasis; and 4. outcome information for overall survival (OS), progression-free survival (PFS) or reports of major complications. We excluded studies that did not provide original data, such as abstracts, case reports, expert opinions, editorials, reviews and letters. Those including treatment with combined or other therapies were also excluded.

### Data extraction and quality assessment

Data were extracted by two independent reviewers (D.P. Y and B.W. Z) using standard forms. The data abstracted from eligible full-text articles included surname of the first author, country of the study population, number of patients, sex, age, HBsAg, tumor size, tumor number, Child-Pugh class, alpha-fetoprotein (AFP) levels, OS, PFS and major complications.

The Newcastle-Ottawa Scale (NOS) was used to assess the quality of the included studies. Scoring was performed by two independent researchers (Y. W and X.Y. X); if a consensus could not be reached, discrepancies were resolved through discussion. The studies were scored on a scale of 0–9 points to quantify the quality of each study. Studies that scored more than 8 points were considered to be of high quality. Studies that scored 6–7 points were classified as having medium quality, while studies with scores below 6 were classified as having low quality [24].

### Definitions

OS was defined as the length of time between the start of treatment for the first RHCC to the date of death related to the tumor or the censoring date if the patients were still alive. PFS was defined as the period from the time of initial recurrence to the date of the second recurrence or death related to the tumor. Treatment-related complications were categorized using the Clavien–Dindo scale [25]. Major complications were extracted according to the data obtained from the included studies.

### Statistical analysis

In the meta-analysis, hazard ratios (HRs) with 95% CIs were calculated for the comparisons of OS and PFS. Some OS or PFS data were indirectly obtained from survival curves [5, 15, 20, 21, 26]. The results of major complications were compared by calculating the odds ratios (ORs) with 95% confidence intervals. The heterogeneity among studies was explored by using the X^2^ test and I^2^ statistic. A *P* value less than 0.05 or I^2^ greater than 50% was considered significant heterogeneity. I^2^ < 25% was considered low heterogeneity, I^2^ ≥ 25 and < 50% was considered moderate heterogeneity. A fixed-effects model was used when there was no or low heterogeneity (I^2^ < 25%); otherwise, a random-effects model was used [27]. An observed HR or OR < 1 suggested that the events (deaths, recurrences, and complications) were more likely to occur in the RHR group than in the RFA group. Sensitivity analysis was conducted by omitting one study at a time to test the influence of individual studies on the pooled estimates. Subgroup analyses were performed in the Chinese population, single RHCC ≤3 cm and single RHCC > 3 cm and ≤ 5 cm. Publication bias was evaluated by Egger’s and Begg’s tests. Significant publication bias was defined as *P* < 0.05. All analyses were performed by Stata 14.0 (Stata Corp., College Station, TX, USA).

## Results

### Study selection

**Fig. 1:**
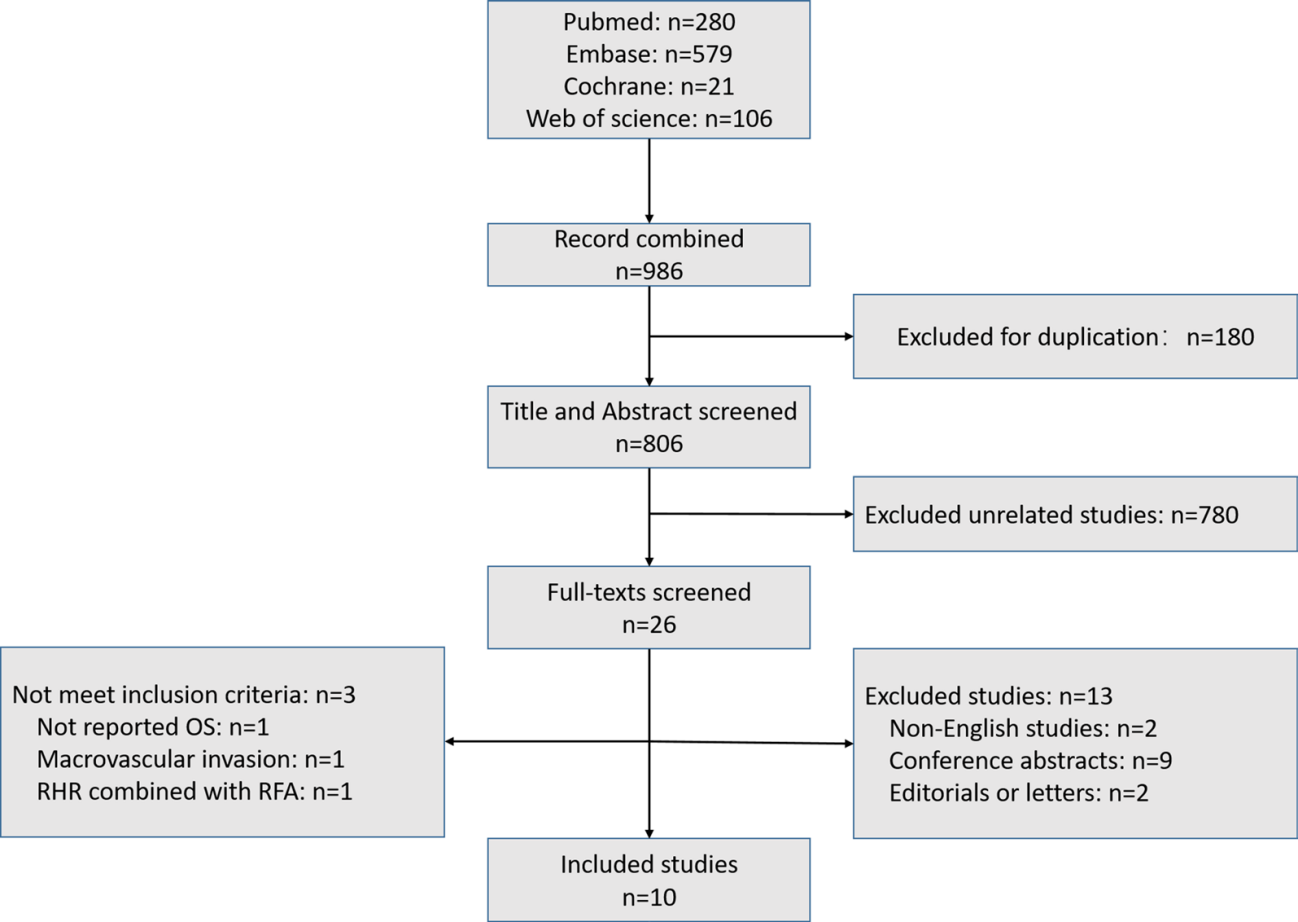
Flow chart for article screening

As shown in Fig. 1, a total of 986 studies were initially obtained, of which 180 were duplicates. After reviewing the titles and abstracts, 26 studies were subjected to full text review. Eighteen studies were further excluded for the following reasons: non-English studies (*n* = 2), conference abstracts (*n* = 9), editorials or letters (*n* = 2), not meeting the inclusion criteria (*n* = 3). Finally, 10 studies with 1612 patients were included in this meta-analysis.

The 10 studies were published between 2008 and 2020. Among the 10 studies, seven were from China, and the rest were from South Korea, Japan and Germany. Most studies were cohort studies, and only one study was an RCT. According to the NOS assessment, the quality scores of the studies ranged from 4 to 9. Of the included studies, 7 were of high quality, 2 were of medium quality and 1 was of low quality. General information of the included studies is summarized in Table 1.

**Table 1:** Baseline characteristics of included studies in the meta-analysis

Study	Treatment	Country	Number of patients	Gender (M/F)	Age, year	HBsAg (+)	Tumor size (cm)	Tumor number	Child-Pugh class (A/B)	AFP(ng/mL)	OS (1/3/5 years) %	PFS(1/3/5 years) %	Major complication
Chan (2012) [5]	RHR RFA	China	29 45	–	52 (38–79)^d^ 59 (36–80)^d^	26 40	2.1 (0.8–5.5)^d^ 2.2 (0.8–6.0)^d^	–	29/0 40/5	64 (2–167,138)^d^ 90 (1–197,122)^d^	89.7/56.5/35.2 83.7/43.1/29.1	41.4/24.2/24.2 32.2/12.4/9.3	3 (10.3%) 1 (2.2%)
Eisele (2013) [26]	RHR RFA	Germany	27 27	15/12 20/7	60 ± 17^e^ 68 ± 7^e^	–	4.0 ± 2.3^e^ 2.8 ± 1.1^e^	16/11^a^ 15/12 ^a^	–	–	100/68/39 96/62/32	82/45/28 51/30/11	4 (14.8%) 1 (3.7%)
Liang (2008) [15]	RHR RFA	China	44 66	41/3 60/6	48.8 ± 12.0^e^ 54.6 ± 10.8^e^	41 60	≦3/>3:26/18 ≦3/>3:44/22	34/10^a^ 48/18^a^	44/0 64/2	≦400/>400: 30/14 ≦400/>400: 52/14	78.6/44.5/27.6 76.6/48.6/39.9	–	30 (68.2%) 2 (3.0%)
Lu (2020) [18]	RHR RFA	China	138 194	31/8 58/20	50.1 ± 10.9^e^ 52.9 ± 11.8^e^	126 172	2.4 ± 1.1^e^ 2.2 ± 1.0^e^	112/26^a^ 162/32^a^	138/0 194/0	≦20/>20:95/43 ≦20/>20:123/71	90.5/81.5/71.8^c^ 91.0/61.0/41.7^c^	–	10 (7.2%) 5 (2.6%)
Song (2015) [16]	RHR RFA	Korea	39 178	31/8 58/20	52.5 ± 9.8^e^ 55.4 ± 10.6^e^	36 149	>2: 17 >2: 50	7^b^ 22 ^b^	39/0 172/6	≦200/>200: 33/6 ≦200/>200: 164/14	88.8/88.8/83.9^c^ 98.7/85.7/72.1^c^	71.8/45.1/39.4^c^ 66.1/48.5/43.1^c^	4 (10.3%) 4 (2.2%)
Sun (2017) [20]	RHR RFA	China	43 57	34/9 38/19	63 (37–84)^d^ 65 (31–84)^d^	21 32	1.9 (0.8–3.0)^d^ 1.8 (1.0–3.0)^d^	1.2 (1–3) 1.1 (1–2)	35/1 50/0	23 (3–290)^d^ 67 (2–817)^d^	97.6/82.7/56.4 98.2/77.2/52.6	57.0/32.1/28.6 60.8/26.6/16.6	1 (2.3%) 0
Umeda (2011) [28]	RHR RFA	Japan	29 58	–	≧65: 16 ≧65: 37	8 11	3.2 ± 0.57^e^ 2.1 ± 0.3^e^	≧3: 4 ≧3: 10	29/0 51/7	≧100: 4 ≧100: 3	93.1/66.8/56.1 94.7/75.1/48.3	–	–
Wang (2015) [17]	RHR RFA	China	128 162	113/15 148/14	50.2 ± 10.1^e^ 52.7 ± 10.9^e^	119 142	2.4 ± 0.9^e^ 2.3 ± 0.7^e^	89/39^a^ 107/55^a^	–	≦20/>20:72/56 ≦20/>20:85/77	97.70/84.1/64.5 96.9/73.4/37.0	–	–
Xia (2020) [22]	RHR RFA	China	120 120	–	–	–	–	–	–	–	92.5/65.8/43.6 87.5/52.5/38.5	85.0/52.4/36.2 74.2/41.7/30.2	7 (5.8%) 2 (1.7%)
Yin (2019) [21]	RHR RFA	China	57 51	41/16 31/20	57.1 ± 12.0^e^ 60.3 ± 9.5^e^	53 48	3.2 ± 2.5^e^ 2.6 ± 0.9^e^	52/5^a^ 48/3^a^	55/2 46/5	167.97 ± 357.23^e^ 266.32 ± 420.28^e^	78.9/50.5/29.7 80.3/50.9/26.0	68.40/39.4/26.6 62.8/32.8/20.4	–

*RHR* repeat hepatic resection, *RFA* radiofrequency ablation, *M* male, *F* Female

^a^single/multiple

^b^multiple

^c^Data extraction after propensity score matching method

^d^Data are presented as median and range in brackets

^e^Data are presented as mean or mean ± standard deviation

### Study characteristics

Of the included patients, 654 underwent RHR, and 958 underwent RFA. The baseline characteristics of the patients, including sex, age, HBsAg(+), tumor size, tumor number, Child-Pugh class and AFP, were homogeneous (Table 1). All of the studies reported 1-, 3- and 5-year OS rates [5, 15–18, 20–22, 26, 28], while only 6 studies [5, 16, 20–22, 26] reported 1-, 3- and 5-year PFS rates. Major complications were reported in 7 studies [5, 15, 16, 18, 20, 22, 26]. The characteristics of the eligible studies are shown in Table 2.

**Table 2:** Results of quality assessment by Newcastle-Ottawa Scale

Study	Selection				Comparability		Assessment of outcome			Total score
	Assignment for treatment	Representative treated arm	Representative reference arm	Demonstration of outcome	Comparable for treated arm	Comparable for reference arm	Assessment of outcome	Adequacy follow up	Acceptable length of follow-up	
Chan [5]	*	*		*	*		*	*	*	7
Eisele [26]	*			*			*	*	*	5
Liang [15]	*	*	*	*	*	*	*	*	*	9
Lu [18]	*	*	*	*	*	*	*	*	*	9
Song [16]	*	*	*	*	*	*	*	*	*	9
Sun [20]	*	*	*	*	*		*	*	*	8
Xia [22]	*	*	*	*	*	*	*	*	*	9
Yin [21]	*	*	*	*	*	*	*	*	*	9
Wang [17]	*	*	*	*	*	*	*	*	*	9
Umeda [28]	*			*		*	*	*	*	6

### Overall survival

**Fig. 2:**
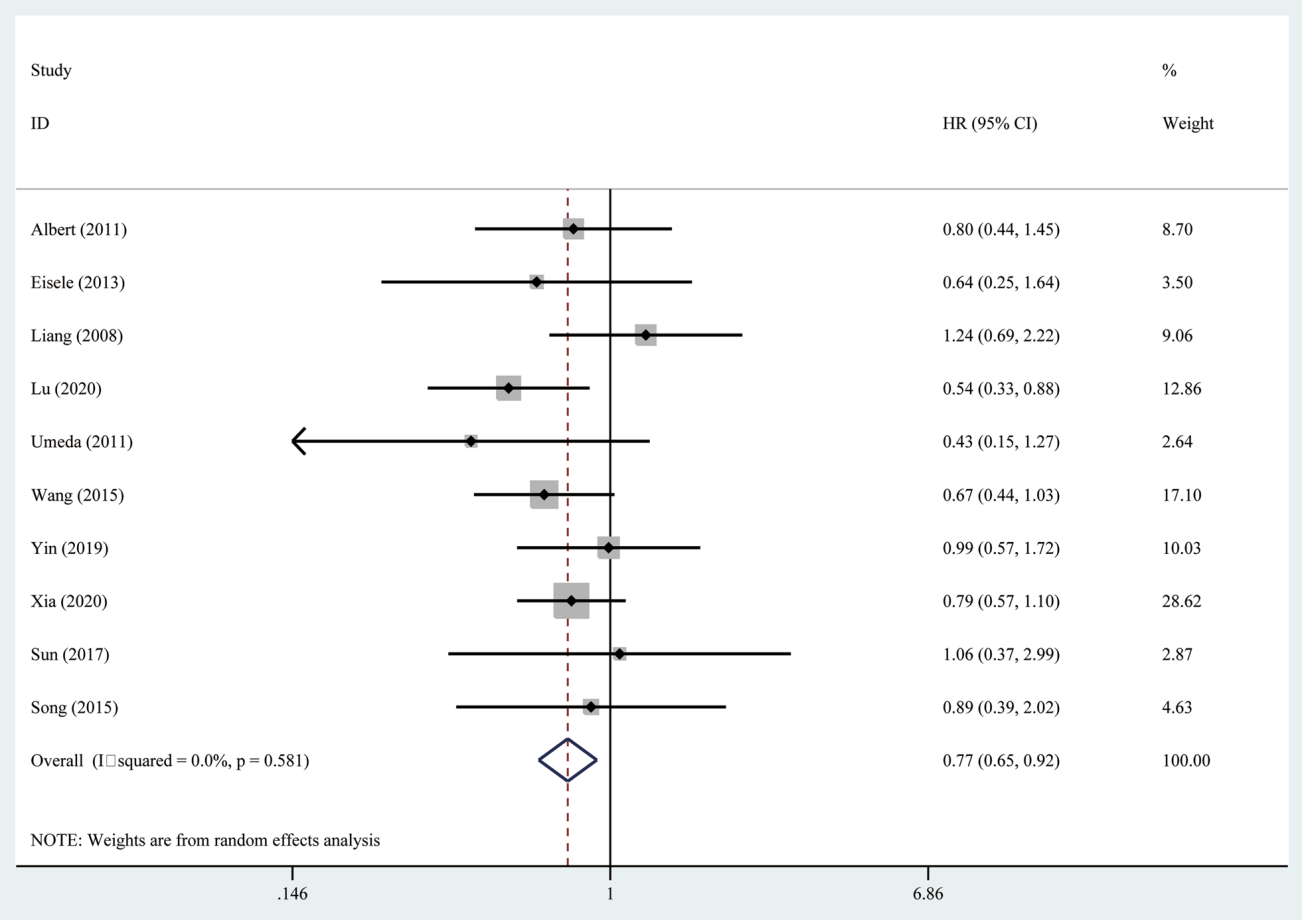
Forest plot for the comparison of Hazard Ratios for overall survival (OS)

The median OS was only reported in 2 of the 10 included studies [22, 26], of which the median OS ranged from 47.1 to 48 months in the RHR group and from 37.5 to 40 months in the RFA group. Meta-analysis showed that the pooled HR was 0.77 (95% CI = 0.65–0.92, *P* = 0.004), and RHR resulted in significantly better OS than RFA. No heterogeneity was detected in the analysis of the effects of overall survival, and a fixed-effects model was used (I^2^ = 0%, *P* = 0.581) (Fig. 2).

### Progression-free survival

**Fig. 3:**
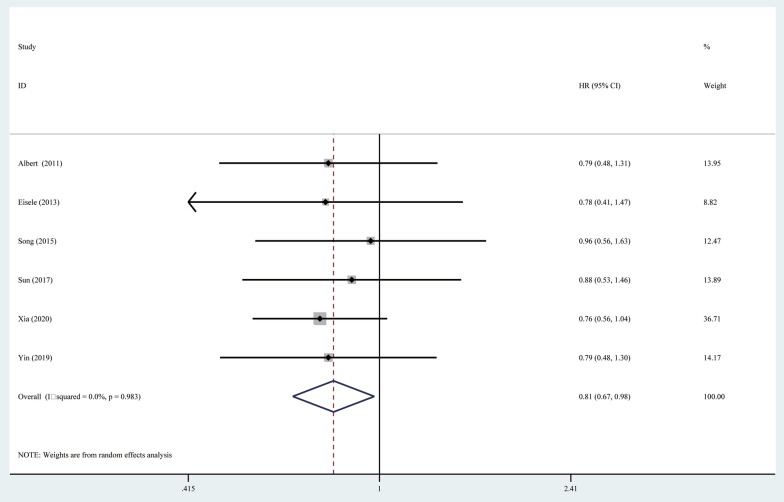
Forest plot for the comparison of Hazard Ratios for progression-free survival (PFS)

Six included studies reported the median PFS. The median PFS ranged from 5.9 to 38.9 months in the RHR group and from 4.0 to 25.8 months in the RFA group. Meta-analysis showed that the pooled HR was 0.81 (95% CI = 0.67–0.98, *P* = 0.027), and RHR provided a PFS advantage for RHCC compared with RFA. The heterogeneity test indicated that a fixed-effects model should be used (I^2^ = 0%, *P* = 0.983) (Fig. 3).

### Complications

**Fig. 4:**
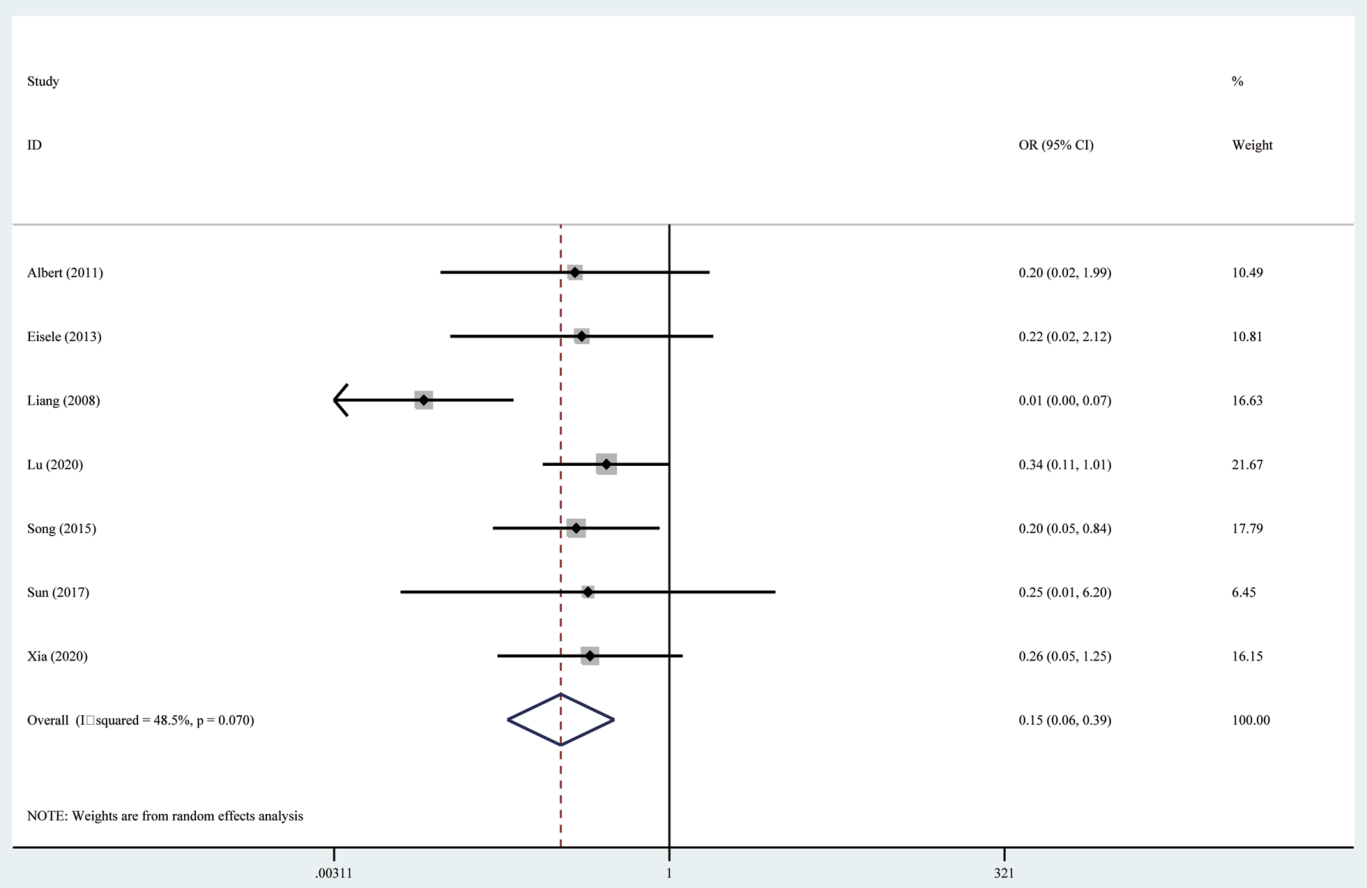
Forest plot for the comparison of Odds Ratios for treatment-related major complication

Major complications in relation to treatment were reported in 7 studies, and the heterogeneity was statistically significant (I^2^ = 49.8%, *P* = 0.063), so a random-effects model was used. Among the 7 studies providing information on major complications, the RHR group had significantly higher rates of major complications than the RFA group (OR 0.15, 95% CI = 0.06–0.39, *P* < 0.001) (Fig. 4). To evaluate the heterogeneity of the meta-analysis, a sensitivity analysis was conducted. The sensitivity analysis showed that the study conducted by Liang et al. [15] significantly affected the heterogeneity of the meta-analysis (I^2^ = 0, *P* = 0.995), while the significant difference in major complications was not materially changed (OR 0.26, 95% CI = 0.13–0.51, *P* < 0.001).

### Subgroup analysis

A total of 7 studies comparing RHR and RFA for RHCC in the Chinese population were included in the subgroup analysis. The results of the subgroup analysis demonstrated that the RHR group had better OS rates than the RFA group (HR 0.79, 95% CI =0.65–0.95, *P* = 0.013) without statistically significant heterogeneity (I^2^ = 1.9, *P* = 0.410). Furthermore, the pooled outcome of PFS in this subgroup showed a significant difference between the two treatment approaches, and RHR led to a better PFS (HR 0.79, 95% CI = 0.64–0.98, *P* = 0.029). No significant heterogeneity was observed in the subgroup analysis of PFS in the Chinese population (I^2^ = 0, *P* = 0.971).

Because of the limited number of research studies, only 3 studies were included in the subgroup analysis of single RHCC ≤3 cm. The results of the meta-analysis suggested that RHR and RFA had equivalent effects on OS (HR 1.03, 95% CI =0.69–1.52, *P* = 0.897). In the subgroup analysis of single RHCC > 3 cm and ≤ 5 cm, RFA was associated with worse OS than RHR (HR 0.57, 95% CI = 0.37–0.89, *P* = 0.014). No significant heterogeneity was detected in the subgroup analyses of single RHCC ≤3 cm (I^2^ = 0%, *P* = 0.799) and single RHCC > 3 cm and ≤ 5 cm (I^2^ = 0%, *P* = 0.900). In the subgroup analysis of single RHCC ≤3 cm for PFS, the result was consistent with that for OS. There was no statistically significant difference in the PFS between the 2 groups (HR 0.99, 95% CI = 0.71–1.37, *P* = 0.929), with no evidence of significant heterogeneity (I^2^ = 0%, *P* = 0.563). However, the subgroup analysis of single RHCC > 3 cm and ≤ 5 cm for PFS could not be performed due to the lack of data. Details of the subgroup analyses are shown in Table 3.

**Table 3:** Subgroup analysis of overall survival and progression free survival

Subgroup	No. of studies	Participants			HR (95% CI)	P	Study heterogeneity			Analysis model
		RHR	RFA	Total			Χ^2^	I^2^ (%)	P	
OS										
Patients in China	7	559	695	1254	0.79 (0.65–0.95)	0.013	6.11	1.9	0.410	Fixed
Tumor size≦3 cm	3	207	243	450	1.03 (0.69–1.52)	0.897	0.45	0	0.799	Fixed
Tumor size>3 cm	2	164	188	352	0.57 (0.37–0.89)	0.014	0.02	0	0.900	Fixed
PFS										
Patients in China	4	249	273	522	0.79 (0.64–0.98)	0.029	0.24	0	0.971	Fixed
Tumor size≦3 cm	2	177	171	348	0.99 (0.71–1.37)	0.929	0.34	0	0.563	Fixed

*No*. number, *OS* overall survival, *PFS* progression-free survival

### Publication bias

**Fig. 5:**
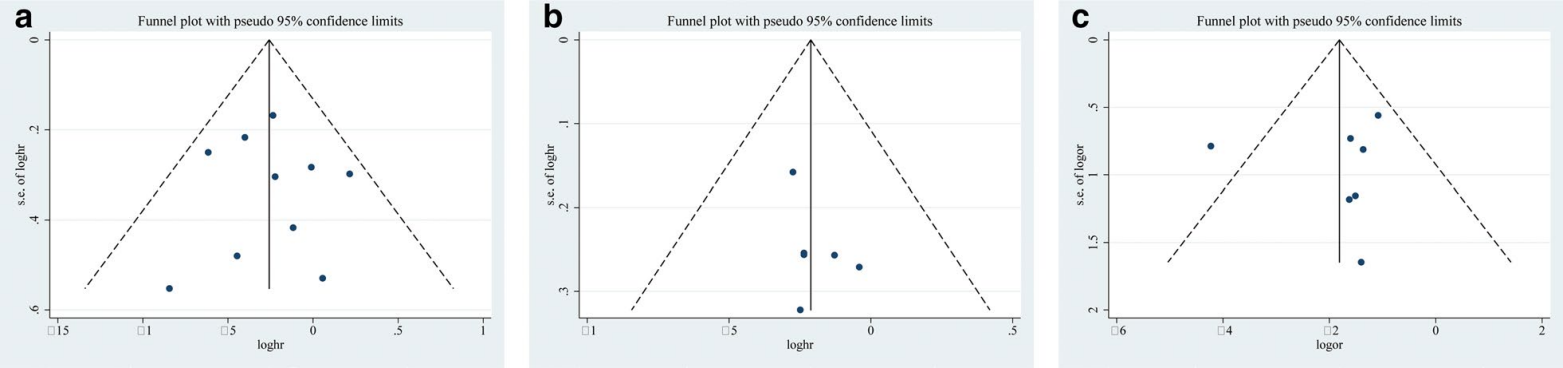
Funnel plot for publication bias. There was no substantial publication bias (**a**) Funnel plot of overall survival and *P* value (calculated with Egger test) of .935 indicates that there was no substantial publication bias). **b** Funnel plot of progression-free survival and *P* value (calculated with Egger test) of .277 indicates that there was no substantial publication bias. **c** Funnel plot of treatment-related major complications and *P* value (calculated with Egger test) of .835 indicates that there was no substantial publication bias

Begg’s and Egger’s tests were applied in the meta-analyses with more than five pooled individual studies. For meta-analyses of OS, PFS and major complications, there was no evidence of significant publication bias upon inspection of the results of formal statistical tests (OS: Egger’s test, *P* = 0.935; Begg’s test, *P* = 0.929; PFS: Egger’s test, *P* = 0.277; Begg’s test, *P* = 0.260; major complications: Egger’s test, *P* = 0.835, Begg’s test, *P* = 0.230; Fig. 5).

## Discussion

This study was a meta-analysis to further evaluate the treatment efficacy and safety of RHR versus RFA in early-stage RHCC. The present study demonstrated that RHR was more effective than RFA for extending the OS and PFS of RHCC patients, especially for patients who had a single tumor > 3 cm and ≤ 5 cm. However, both RHR and RFA were suitable for single RHCC ≤3 cm. Moreover, the results suggested that the incidence of complications was lower for patients treated by RFA. To our knowledge, this study included the largest study population and presents the latest meta-analysis including new studies published within the last 5 years. In addition, a randomized clinical trial was included in the meta-analysis, which contributed to a high evidence level. Therefore, the results of this study can provide important data with which guidelines for the management of RHCC after initial treatment could be established.

Several previous meta-analyses have been performed to evaluate the outcomes of RHR versus RFA for RHCC patients, of which Gavriilidis et al. concluded that the RHR and RFA groups had similar OS and PFS rates [9], while only five retrospective studies were included in the study [5, 15, 16, 26, 29]. On the other hand, another previous meta-analysis showed that RHR was associated with comparable OS rates and higher PFS rates [8, 10, 19]. The differences between the findings of previous meta-analyses and those of our study might be explained by the following reasons. First, in previous studies, the number of included studies was small, and non-RCTs were included in the evaluation. Second, though several studies reported generally comparable outcomes between RHR and RFA, a tendency toward longer OS and PFS was observed in the RHR group compared with the RFA group [22, 26, 28]. Third, two recent high-quality studies revealed that RHR was still the most effective treatment, followed by RFA for RHCC [17, 18], and the results may play an important role in the meta-analysis. However, these two studies had never been included in previous meta-analyses.

According to the outcomes, tumor recurrence may be one of the most important factors affecting OS in patients with RHCC. There are many factors associated with tumor recurrence, and the completeness and safety margin of treatment are key elements. HCC has a tendency to invade portal branches and thus cause tumor dissemination along the liver segment [30]. Segment-based anatomic partial hepatectomy can remove both the primary tumor and microvascular invasion, together with at least 1 cm of the rim of normal hepatic parenchyma [31]. However, in the RFA procedure, it is hard to create a sufficient safety margin precisely in the 3-dimensional liver with the guidance of 2-dimensional ultrasonography [32]. There is also a lack of objective evaluations of the safety margin and ablation effect. In addition, some risk factors for recurrence are associated with RFA but not with resection. For example, difficult locations, such as a tumor located on the liver surface or near the main hepatic vessels or hilum, are a worsening indicator in ablation [33]. Moreover, the complete ablation rate is affected not only by tumor location but also by the experience of the operator. Therefore, it is not surprising that RFA has been frequently reported to have higher recurrence rates than resection for the treatment of HCC [33].

With regard to treatment-related complications, RHR was associated with a greater incidence of major complications than RFA, which should be attributed to the minimally invasive characteristic of RFA. Compared with RHR, RFA can be performed percutaneously, thus greatly minimizing the surgical impact. In addition, RFA preserves as much liver parenchyma as possible and causes minor damage to the remnant liver [34]. Hence, RFA can serve as an alternative choice of treatment for early-stage RHCC with the advantage of less invasiveness.

In the subgroup analysis of patients in China, the results concerning OS and PFS were similar to the outcomes of the meta-analysis without regional restriction. This finding was also confirmed by the study of Chen et al. [10]. According to the latest data, approximately 46.71% of new cases of HCC are diagnosed in China, and over 85% of patients with HCC are linked with hepatitis B virus infection [35]. Therefore, the results of the subgroup analysis add weight to the current clinical decision in the Chinese population.

Another subgroup analysis was performed in RHCC ≤3 cm, of which RFA achieved equivalent OS and PFS rates compared with RHR. After initial resection, RHCC is usually smaller than 3 cm under intensive screening [20]. Previous studies have demonstrated that a smaller tumor size is closely related to an increased chance of complete ablation [36]. This may be because RFA can achieve a greater safety margin than RHR for RHCC ≤3 cm. As expected, subgroup analyses demonstrated better OS after RHR than after RFA among patients with an RHCC diameter greater than 3 cm. Unfortunately, these results should be further explored because of the limited number of included research studies in the subgroup analysis.

RHR was considered if patient had a single tumor or oligonodular tumor within a monosegment of liver when there was the possibility for the complete removal of all tumors while retaining a sufficient liver remnant [16]. However, the reported rate of RHR for RHCC in clinical practice was less than 30% [37]. As an effective alternative for surgery, RFA has some advantages when compared with RHR in treating RHCC. First, as a minimally invasive treatment modality, RFA can greatly decrease the incidence of major complications. Second, repeatability is a major advantage of RFA [5]. For patients with limited liver remnants, RFA may serve as an ideal treatment choice. Therefore, for those who are unsuitable for RHR or have a tumor size smaller than 3 cm, RFA may be a replacement therapy for resection because of its safety and feasibility.

Moderate heterogeneity was found in the meta-analysis of major complications. Sensitivity analysis was conducted by eliminating each study in turn. Finally, we found that the heterogeneity of the meta-analysis mainly came from the study of Liang et al. [15]. In the study of Liang et al., major complications were defined as complications with Clavien–Dindo classification grade II or higher, while in the other included studies, grade III or higher was applied [5, 16–18, 20–22, 26, 28]. Consequently, the definition in Liang’s study overestimated the incidence of major complications, which led to heterogeneity.

This meta-analysis has several limitations. First, only a small number of studies examined the treatment options for RHCC. A total of 10 studies were included in this meta-analysis, and only 6 reported PFS. Second, indirect data acquisition obtained from survival curves may have an effect on our outcomes. Third, only 2 or 3 studies in the subgroup analysis covered the tumor size of RHCC, and more evidence is needed in future studies. Furthermore, many studies have demonstrated that the number of lesions and Child-Pugh class are important prognostic factors [38, 39]. Also, many other confounders including receiving anti-viral agent, progression or occurrence of cirrhosis, the duration of the procedure and the expertise of care team may effect on the overall survival and outcome. However, the data on these confounders were not sufficient for meta-analysis in subgroups.

## Conclusion

The current available evidence demonstrates that RHR provides better outcomes than RFA for RHCC, especially in patients who have a single RHCC > 3 cm and ≤ 5 cm. For patients with single RHCC ≤3 cm, RFA provides comparable benefits to RHR in OS and PFS with lower complications in patients with early-stage RHCC. M ore multicenter RCTs with strict selection criteria and a greater number of included patients are needed to provide reliable evidence for the long-term efficacy of the two treatment arms.

## Supplementary Information

**Additional file 1.** PRISMA 2009 Checklist.

**Additional file 2.** Search Strategy.

## Data Availability

The datasets used and/or analysed during the current study available from the corresponding author on reasonable request.

## Abbreviations

RFA: Radiofrequency ablation
RHR: Repeat hepatic resection
RHCC: Recurrent hepatocellular carcinoma
OS: Overall survival
PFS: Progression-free survival
HR: Hazard ratio
OR: Odd ratio
HCC: Hepatocellular carcinoma
RCTs: Randomized controlled trials
PRISMA: Preferred Reporting Items for Systematic Reviews and Meta-Analyses
PROSPERO: Prospective Register of Systematic Reviews
AFP: Alpha-fetoprotein
NOS: Newcastle-Ottawa Scale
