# Supplementary material for: Radiofrequency ablation versus hepatic resection for recurrent hepatocellular carcinoma: an updated meta-analysis
Source: BMC Gastroenterol. 2020 Nov 27;20:402. doi: 10.1186/s12876-020-01544-0 (PMC7693504; doi:10.1186/s12876-020-01544-0)
Supplement: Supplementary file 2 — Additional file 2. Search Strategy. [file 12876_2020_1544_MOESM2_ESM.docx]

Search strategy

Pubmed: n=280

(((((((radiofrequency ablation[Title/Abstract]) OR (Ablation, Radiofrequency[Title/Abstract])) OR (Radio Frequency Ablation[Title/Abstract])) OR (Ablation, Radio Frequency[Title/Abstract])) OR (Radio-Frequency Ablation[Title/Abstract])) OR (Ablation, Radio-Frequency[Title/Abstract])) AND (((liver resection[Title/Abstract]) OR (hepatic resection[Title/Abstract])) OR (hepatectomy[Title/Abstract]))) AND ((((((Recurrent[Title/Abstract]) ) OR (recurrence[Title/Abstract])) OR (relapse[Title/Abstract])) OR (recurring[Title/Abstract])) AND ((((((((((((((((((Carcinoma, Hepatocellular[Title/Abstract]) OR (Carcinomas, Hepatocellular[Title/Abstract])) OR (Hepatocellular Carcinomas[Title/Abstract])) OR (Liver Cell Carcinoma[Title/Abstract])) OR (Adult Liver Cancer[Title/Abstract])) OR (Adult Liver Cancers[Title/Abstract])) OR (Cancer, Adult Liver[Title/Abstract])) OR (Cancers, Adult Liver[Title/Abstract])) OR (Liver Cancers, Adult[Title/Abstract])) OR (Liver Cell Carcinoma[Title/Abstract])) OR (Carcinoma, Liver Cell[Title/Abstract])) OR (Carcinomas, Liver Cell[Title/Abstract])) OR (Cell Carcinoma, Liver[Title/Abstract])) OR (Cell Carcinomas, Liver[Title/Abstract])) OR (Liver Cell Carcinomas[Title/Abstract])) OR (Hepatocellular Carcinoma[Title/Abstract])) OR (Hepatoma[Title/Abstract])) OR (Hepatomas[Title/Abstract])))

Cochrane Library: n=21

“Recurrent or recurrence or relapse or recurring” in Title, Abstract, Keywords and “Liver Cell Carcinoma or Hepatomas or Liver Cancers, Adult or Liver Cell Carcinoma, Adult or Adult Liver Cancers or Carcinomas, Hepatocellular or Cancer, Adult Liver or Carcinoma, Liver Cell or Cancers, Adult Liver or Cell Carcinomas, Liver or Hepatocellular Carcinoma or Liver Cancer, Adult or Hepatocellular Carcinomas or Adult Liver Cancer or Cell Carcinoma, Liver or Liver Cell Carcinomas or Hepatoma or Carcinomas, Liver Cell” in Title, Abstract, Keywords and “Radio-Frequency Ablation or Ablation, Radiofrequency or Radio Frequency Ablation or Ablation, Radio Frequency or Ablation, Radio-Frequency in Title, Abstract, Keywords” and “liver resection or hepatectomy or hepatic resection” in Title, Abstract, Keywords.

Embase: n=579

Topic: (Recurrent or recurrence or relapse or recurring) AND (liver cell carcinoma carcinoma hepatocellular or carcinoma, hepatic cell or carcinoma, liver or carcinoma, liver cell or hepatic carcinoma or hepatic cell carcinoma or hepatocarcinoma or hepatocellular carcinoma or hepatoma or liver carcinoma or liver carcinoma rupture or malignant hepatoma or primary liver carcinoma) AND (Radiofrequency ablation or

radio frequency ablation or RFA (radiofrequency ablation) or RFA therapy) AND (liver resection or hepatectomy or hepatic resection or resection, liver)

Web of science: n=106

Topic: Recurrent AND Topic: (((((((((((((liver cell carcinoma or carcinoma, hepatocellular) or carcinoma, hepatic cell) or carcinoma, liver) or carcinoma, liver cell) or hepatic carcinoma) or hepatic cell carcinoma) or hepatocarcinoma) or hepatocellular carcinoma) or hepatoma) or liver carcinoma) or liver carcinoma rupture) or malignant hepatoma) or primary liver carcinoma)) AND Topic: (((((radiofrequency ablation or Ablation, Radiofrequency) or Radio Frequency Ablation) or Ablation, Radio Frequency) or Radio-Frequency Ablation) or Ablation, Radio-Frequency)) AND Topic: ((liver resection or hepatic resection) or hepatectomy))
